# Editor’s Book Table

**Published:** 1869-12

**Authors:** 


### Editor's Book Table.

[NOTE.—All works reviewed in the columns of the CHICAGO MEDICAL JOURNAL may be found in the extensive stock of W. B. Keen & Cooke, whose catalogue of medical books will be sent to any address upon request.]

*A Treatise on Intraocular Tumors.* From Original Clinical Observations and Anatomical Investigations. With one Chromo-Lithographic and Fifteen Lithographic Plates, containing very many Figures. By H. KNAPP, M.D., late Professor of Ophthalmology, and Surgeon to the Ophthalmic Hospital in Heidelberg. Translated by S. Cole, M.D., of Chicago: New York: William Wood & Co., 61 Walker Street. 1869. Pp. 323.

THE translator of this essay will be recognized by readers of the JOURNAL as the author of several very interesting letters for its pages, during his residence in Europe, and while in attendance upon Dr. Knapp's clinical and didactic course at Heidelberg. As his attendant, Dr. Cole saw most of Dr. K.'s anatomical preparations, and observed some of the cases described during life. He is, therefore, as Dr. K. remarks in the preface to the English edition, thoroughly conversant with the subject; a circumstance no less indispensable for a good translator than a perfect knowledge of both languages.

In his pathology, the author adopts mainly the views of Virchow in his work on Morbid Tumors, but observes: "If I was to rely upon the results obtained from my own observation, ocular tumors would hardly admit of more than two varieties, viz.: "*Glioma*, originating in the *retina;* and *sarcoma*, proceeding from the choroid, and being in part *unpigmented* and in part *melanotic*."

The descriptions, both general and particular, are very clear and explicit, and constitute a valuable contribution to ophthalmology.

The plates are beautifully executed, and the explanations very full and complete. We congratulate the author on the excellence of the translation, and general typographical appearance of the book. The ophthalmologists, just now, seem to be outstripping all other specialists in the production of new and elegant volumes.

*Diseases and Injuries of the Eye; their Medical and Surgical Treatment.* By George Lawson, F.R.C.S., Surgeon to the Royal London Ophthalmic Hospital, Moorfields, and Assistant Surgeon to the Middlesex Hospital. Philadelphia: Lindsay & Blakiston. 1869. Pp. 436.

This book is an entirely distinct one from the treatise on "Injuries of the Eye, Orbit and Eyelids," by the same author, an edition of which was published by Mr. H. C. Lea in 1867. Those who are familiar with that work, will be prepared to receive the present one with pleasure. It is not intended to take the place of the more voluminous treatises on the eye, but to afford the practitioner a concise account of modern ophthalmological science. It has evidently been carefully written, and in addition to the writer's own experience, gives frequent reference to the practice of his colleagues at the Royal London Ophthalmic Hospital, and the labors of the continental ophthalmic surgeons.

A formulary of prescriptions, a page of test-types (Dr. Pray's), and a copious index add materially to the value of the book.

*A Compend of Materia Medica and Therapeutics, for the use of Students.* By John C. Riley, A.M., M.D., Professor of Materia Medica and Therapeutics in the National Medical College; one of the Physicians of Providence Hospital, Washington, D. C., etc., etc. Philadelphia: J. B. Lippincott & Co. 1869. Pp. 370.

A "comprehensive syllabus," with outline descriptions, adopting the classifications usually employed in the lecture-room, and the formularies, physical histories, etc., given in the dispensatories and larger systematic works. From the casual examination we have had time to give it, we judge it a convenient and reliable manual.

*Percussion and Auscultation as Diagnostic Aids. A Manual for Students and Practitioners of Medicine.* By Dr. CARL HOPPE, Assistant Physician to the Sixth Westphalian Regiment of Infantry. Translated by L. C. Lane, M.D. Philadelphia: J. B. Lippincott & Co., 1869. Pp. 152.

THE author herein seeks to give in a condensed form the views of Scoda, Piorry, Traube, Wintrich and Bamberger. Especial attention is given to the views of Traube. Although claiming to be nothing more than a compend, we fully coincide with the translator's remark, that "it contains far more than many of the large works on the same subject—being, in fact, a master piece, both in thoroughness and brevity."

Those who may already have the capital epitome by Barth and Rogers, Loomis, Flint and others, will nevertheless do well to add this to their libraries. The chapter on the Apex-Impulse (appendix) is alone worth the price of the book. It is the only satisfactory *resume* of the topic it has been our fortune to meet.

*Reports of the Illinois State Hospital for the Insane.* 1847 to 1862. Pp. 422.

WE have also to acknowledge herewith several pamphlets (elsewhere noticed) from A. McFarland, M.D., Superintendent.

*A Physician's Problems.* By CHARLES ELAM, M.D., M.R.C.P. Boston: Fields, Osgood & Co., 1869. Pp. 400.

IT is with a sense of unmingled pleasure that, for once, we can chronicle the appearance of a book suitable for popular reading, at least of educated and thinking men. Dr. Elam has collected herein many topics of surpassing interest, and, in the main, has elucidated them in a manner which must command attention and inspire healthful thought.

Although intended for general perusal, we sincerely hope that among his readers the author may secure that large class of physicians, we are happy to believe daily increasing in numbers, who rise above the bald routine of professional practice to a philosophic consideration and study of the grand first principles and large comprehensive truths which entitle Medicine to be ranked, not only as the most useful of arts but as the noblest of sciences.

The "problems" discussed are eight in number, and we regret that we can spare but little space for quotations or even abstracts.

The first problem is that of *Natural Heritage*, and in the exceedingly able and interesting chapter unfolding his views and the facts upon which they rest, he concludes:

1. In procreation, as in creation, we every where trace the operation of two principles, Similarity and Diversity.

2. In obedience to the law of similarity, 'like produces like,' equally in species, in races, and in families.

3. In obedience to the law of diversity, children differ from their parents and from each other. In accordance, also, with this law, there is the power of returning to the specific type, whatever may have been the modifications produced accidentally, or by the influence of circumstances, upon the race; even as, according to Mr. Darwin, the different varieties of pigeon evince a tendency to return to the 'blue rock' type.

4. We have seen reason to conclude that these two laws are not so much opposed as their names would appear to imply; but that diversity is produced by the very potency of operation of the law of similarity, whereby temporary and accidental conditions are propagated.

5. Every formation of body, internal or external; every deformity or deficiency, from disease or accident; every habit and every aptitude—all these are liable to be, or *may* be, transmitted to the offspring. In the case of accidental defects and modifications of the specific type, the offspring *usually* do not inherit them, but return to the normal type.

6. Intellectual endowments and aptitudes are liable to transmission; and according to the mental cultivation or neglect of the parents will be (as a general rule) the capacity and facility of learning of the children. This will be more evident in proportion to the number of generations through which such cultivation or neglect has been practiced.

7. All moral qualities are transmissible from parent to child, with this important addition, that, in the case of vicious tendencies or habits, the simple practice of the parent becomes the passion, the mania, the all but irresistible impulse of the child.

8. Even where the very identical vice is not inherited, a morbid organization is the result, which shows itself in some allied morbid tendency or some serious physical lesion.

9. All chronic diseases appear to be transmissible, either in the original form, or in the form of a transformation of the morbid tendency.

10. These inheritances, normal or abnormal, are not always immediate from the parents, or even in a direct line; but they miss one or more generations, and sometimes have only appeared in collateral branches, as an uncle or grand uncle, etc. The reason for this may be deduced from what has been stated above. A man, for instance, does not inherit all the qualities of his father or mother; and of those which he does inherit, only some are developed, whilst others remain latent, and are probably developed in a brother or sister. But his son may in turn inherit the same faculties, with this difference, that those which were but latent or potential in the father are fully manifested in him; and so he comes to resemble not his own father (or mother) so much as his uncle or aunt, or some more distant relative, still descended from one common stock.

11. Of all morbid heritages, unsoundness of mind in its numerous forms seems to be the most certain and constant; and the results form a considerable proportion of our criminal population.

12. But whilst by the law of Similarity children become subject to the imperfections of their parents, by the law of Diversity they are enabled to escape from them; these evils are not necessarily entailed, and a proper comprehension of the principles upon which these diversities depend, enables us to take such measures as will facilitate this escape. And whilst on the one hand we see unhealthy children proceeding from healthy parents, on the other we see the morbid tendencies of the parents counteracting each other, and giving rise to a healthy offspring.

13. The offspring of that large portion of our population who are given up to intemperance and other forms of vice inherited from their parents' strong impulse and feeble wills, so as to become more or less irresponsible, bear a peculiar relation to the law—a relation which urgently claims an attention and investigation which it has, as yet, very imperfectly received.

14. It is highly improbable, and perhaps undesirable, that matrimonial unions should ever be formed on the scientific principle which would lead to the fulfillment of the possibilities hinted at in the foregoing observations; yet a due consideration of such principles may be serviceable in avoiding glaring and palpable evils, if not in producing the actual benefit which *might* accrue under other arrangements.

The second problem is: *How are our armies of Crime and Disease recruited?* and treated under the head of Degenerations in Man.

Each race of men look back to the type of perfection, the original man, as a countryman of their own, and in fact we have no absolute standard of comparison. The author does not believe in the Development doctrine in any wise.

"Even had we no revelation, the hypothesis of a fall from a previous higher state, through the action of various climatic and moral agencies, would present infinitely fewer difficulties in accounting for the phenomena than this absurd theory."

The mode in which the *varieties of mankind* both physical and moral are produced, is gathered from the considerations set forth in the chapter on Natural Heritage.

Natural modifications of the primitive type occur from ordinary climatic and other terrestrial agencies. Morbid deviations from this type may occur from varying circumstances attendant upon his nutrition, his social condition, his habits of life, hereditary influences, etc. This constitutes a *degeneration*, and is characterized by a tendency to further deterioration, to hereditary transmission, and to the more or less speedy extinction of that section of the race or community. Mental alienation (*moral*) in its various forms, but especially the chronic, is but the concentrated and final expression of degeneracy of race, wheresoever the chain of morbid phenomena commenced.

"Observation shows that, failing certain exceptional elements of regeneration, the offspring of degenerate beings present types of *progressive* degradation. This progression may attain such limits that humanity is only preserved by the *very excess of the evil*, and the reason is plain: the existence of degenerate beings is necessarily bounded, and it is not even necessary that they should reach the *last degree* of degeneracy in order to be smitten by sterility, and become incapable of transmitting the type of their degradation."

Acclimation in individuals may not pass the boundary of natural modification, yet often produces actual degeneration.

"Between the intellectual state of the most savage Bosjesman and that of the most refined European, there is much less difference than between the state of the said European and that of the *degenerate* being whose intellectual arrest is due to cerebralatrophy, congenital or acquired, or to any morbid influence leading to imbecility, idiocy, or dementia."

Natural modification is susceptible of some radical improvement, but the morbid deviation is only susceptible of relative amelioration, and a fatal heritage will descend to the progeny.

"There exist certain individuals who resume in their own persons the morbid organic tendencies of many previous generations."

"Asylums for the insane are, in this view, but a concentration of the principal forms of degeneration in the race."

Many of the principal probable causes of degeneration, *now* in operation, are suggested in the chapter, which deserve careful reflection, and this very sensible view is taken:

"Whether the human race, as a whole, is in a state of degeneration or not, is not the question; perhaps were it so, it might not be an insoluble one. But it is clearly proved, or provable, that a great number of special degenerations are in progress in the species, and that these are in certain proportion to well-defined causes. These causes are in some degree removable; in other respects, owing to the constitution of society, they admit only of more or less modification. Be this as it may, the first step in the process is to *point out the source* of these evils, and the mode in which they first act upon individuals, and, through them, upon society at large."

The third problem refers to moral and criminal epidemics:

*Are mental affections and tendencies contagious, like bodily diseases? If so, under what conditions?*

The stand-point is this: "The philosopher recognizes no accident. To him there is no phenomenon without *a cause*, an antecedent adequate to its production; no cause but such as is reducible to *law*."

The elements are constant, though their combinations may be variable. The *history* of yesterday is the interpretation of *to-day*, the *prophecy* of to-morrow. The *irregularities* of the pendulum determined for us the shape of the earth, and the *irregularities* of Saturn and Uranus brought to light a planet where it ought to be. The *casualties* of polarized light defined and advanced all the laws of optics.

Thus the prevalence of crimes, delusions, and all forms of so-called aberrations of human thought and action are found to be referrible to known or ascertainable laws. "Man, who has an *individual* responsibility, is the plaything not only of *his own* passions and instincts, but, through the laws of his being, also of those of others."

The fourth problem is:

*What effect has the work of the brain upon life, health and mind?*

The writer receives as a philosophical truth that mind is a distinct entity—something independent of, and distinct from, matter; closely united, yet not allied; dependent for its manifestation, yet distinct in essence. An idea was prevalent among the philosophers of antiquity that the body was a clog and impediment to the acquisition of knowledge, distracting attention by its material relations and requirements; and later theologians look upon it as sin incarnate, the source of all evil and temptation, the barrier between the soul and heaven. In our times this assumed antagonism is merging in wider views.

"The present is essentially a practical age; both strong limbs and thoughtful minds are in requisition; and the spirit of the age is in nothing more manifest than in the multiform attempts, by the spread of rational education, and the increased attention to the sanitary condition of the masses, to balance the interests of these hitherto conflicting elements."

The brain is to be considered as perfectly adapted to the performance of its functions — thought, perception, volition, etc., as the muscles are for their offices; alike requiring, for perfection of development, appropriate nutrition, exercise and rest. It is strengthened and perfected by rational use, and injured by excess, just as other organs are.

Most of the evils attributed to mental labor more probably arise from the same causes which operate in other sedentary occupations. The youth of feeble and degenerate organization is selected as unfitted for physical labor, and doomed to the cramped position, the confined atmosphere, and generally unhealthy habits of the student; or the precocity of disease, often mistaken for genius or mental power, is forced by hot-house pressure. "The life is short, not *because* the intellectual development is precocious or forced, but it is short and bright from a common cause deeply ingrained in the original exceptional organization."

The case is, if possible, worse when a boy or man of talents below mediocrity is ambitious to excel in pursuits for which he is unfitted — as cripples may be anxious to excel in running or jumping. There is no more melancholy spectacle than a person mistaking their love for any object of mental effort for talent, and wearing out their lives in hopeless efforts at performance —

"Ever failing, yet sometimes happily unconscious of the failure; trying again and again, yet ever again coming far short of even their own imperfect ideal, — finally succumbing, worn out by constant attrition against the rock of the impossible."

"There are two sorts of good constitutions — good idle constitutions, and good working ones. When nature makes a great man, she presents him the latter gift."

Mr. Maclaren, of the Gymnasium, Oxford, says: "In the acquirement of bodily health, *mental occupation is a helpful, indeed, a necessary agent.*"

All reliable statistics show that *mental athletes* surpass, by far, in longevity mere *physical* athletes. He concludes:

"The injurious effects of mental labor are, in a great measure, owing—
To excessive forcing in youth;
To sudden or misdirected study;
To the co-operation of depressing emotions or passions;
To the neglect of the ordinary rules of hygiene;
To the neglect of the habits of the body; or,
To the presence of the seeds of disease, degeneration and decay in the system."

Illusions and hallucinations afford problem fifth:

*Under what conditions are our senses reliable or unreliable witnesses?*

An illusion is *a false appreciation of a real sensation.* A hallucination is *a projection externally of an inward conception;* in other words, *a subjective sensation.* This section, and its appendix, concerning Socrates and Pascal, are deeply interesting and instructive.

Somnambulism gives the problem:

"*To what extent may we be made the unconscious playthings of our physical organization?*"

*Reverie and abstraction* suggest the last problem:

"*To what extent is attention entitled to be considered the one great working faculty of the mind?*"

A great book is often a great mischief, and a little book is, now and then, as full of good things "as an egg is of meat." This little book, in our opinion, comes in the latter category — but we have given it all the space we can spare, and must close the notice "without ceremony."

